# Low occurrence of *Pseudomonas aeruginosa* in agricultural soils with and without organic amendment

**DOI:** 10.3389/fcimb.2014.00053

**Published:** 2014-04-29

**Authors:** Amélie Deredjian, Céline Colinon, Edmond Hien, Elisabeth Brothier, Benjamin Youenou, Benoit Cournoyer, Samuel Dequiedt, Alain Hartmann, Claudy Jolivet, Sabine Houot, Lionel Ranjard, Nicolas P. A. Saby, Sylvie Nazaret

**Affiliations:** ^1^CNRS, Ecole Nationale Vétérinaire de Lyon, and Université Lyon 1, UMR 5557 Ecologie Microbienne, Université de LyonVilleurbanne, France; ^2^UMR Ecosol, IRD-Université de Ouagadougou, UFR/SVTOuagadougou, Burkina Faso; ^3^Plateforme GenoSol, INRA-Université Bourgogne, AgroSup, UMR1347 AgroécologieDijon, France; ^4^INRA-Université Bourgogne, AgroSup, UMR1347 AgroécologieDijon, France; ^5^INRA, US 1106 InfoSolOrléans, France; ^6^INRA, UMR 1091 Environnement et Grandes CulturesThiverval-Grignon, France

**Keywords:** *Pseudomonas aeruginosa*, qPCR, cultivation, soil, organic amendment

## Abstract

The occurrence of *Pseudomonas aeruginosa* was monitored at a broad spatial scale in French agricultural soils, from various soil types and under various land uses to evaluate the ability of soil to be a natural habitat for that species. To appreciate the impact of agricultural practices on the potential dispersion of *P. aeruginosa*, we further investigated the impact of organic amendment at experimental sites in France and Burkina Faso. A real-time quantitative PCR (qPCR) approach was used to analyze a set of 380 samples selected within the French RMQS (“Réseau de Mesures de la Qualité des Sols”) soil library. In parallel, a culture-dependent approach was tested on a subset of samples. The results showed that *P. aeruginosa* was very rarely detected suggesting a sporadic presence of this bacterium in soils from France and Burkina Faso, whatever the structural and physico-chemical characteristics or climate. When we analyzed the impact of organic amendment on the prevalence of *P. aeruginosa*, we found that even if it was detectable in various manures (at levels from 10^3^ to 10^5^ CFU or DNA targets (g drywt)^−1^ of sample), it was hardly ever detected in the corresponding soils, which raises questions about its survival. The only case reports were from a vineyard soil amended with a compost of mushroom manure in Burgundy, and a few samples from two fields amended with raw urban wastes in the sub-urban area of Ouagadougou, Burkina Faso. In these soils the levels of culturable cells were below 10 CFU (g drywt)^−1^.

## Introduction

*Pseudomonas aeruginosa* is a ubiquitous Gram-negative bacillus and an opportunistic pathogen considered as one of the major agents of nosocomial infections. It can cause infections among patients with weakened defense barriers, such as severely burned or artificially ventilated patients (Richard et al., 1994), and is the main pathogen associated with respiratory tract infection in cystic fibrosis patients (Saiman and Siegel, 2004). Infections in healthy individuals can occur as keratitis (Song et al., 2000), otitis (Heslop and Ovesen, 2006), and others. *P. aeruginosa* can also be detected in human and animal fecal samples (Mushin and Ziv, 1973; Lavenir et al., 2008). It has been identified as an animal pathogen responsible for various infections (Daly et al., 1999; Ledbetter et al., 2007). The global increase of the worldwide population, combined with the increasing numbers of patients at risk might then favor the incidence of infection by this opportunistic pathogen. Knowing more about its ecology and natural reservoirs is therefore important to avoid infections and outbreaks.

This species is intrinsically resistant to a wide range of antimicrobials, has wide metabolic versatility and can be found in wide variety of ecological environments. It is described as preferentially living in aquatic habitats and colonizing moist niches. Within hospital settings, it was isolated from sinks and tubs (Römling et al., 1994), tap water outlets (Reuter et al., 2002), or water pipes (Lavenir et al., 2008). In outdoor environments it was isolated from a wide range of water sources including rivers (Pirnay et al., 2005; Selezska et al., 2012), open ocean (Khan et al., 2007), and can be recovered in high numbers from recreative waters (Barben et al., 2005) or wastewater (Lavenir et al., 2007; Lee et al., 2008).

Considering terrestrial environments, its isolation from plants (Green et al., 1974; Cho et al., 1975) or vegetables (Wright et al., 1976) as well as its detection in agricultural soils (Green et al., 1974; Marques et al., 1979) have been reported. However, whether these environments act as a reservoir or a transient recipient of *P. aeruginosa* is still under debate and data on the factors driving its survival and dissemination are scarce. The oldest report on its occurrence in soils was from Ringen and Drake (1952). Later, Green et al. (1974) recovered it from Californian soils and concluded about agricultural soil as a natural habitat for the bacterium since the soils sampled had no known organic fertilizer or animal pasturing background, and irrigation water was free of the bacterium. Further studies showed that *P. aeruginosa* is often present within polluted soils from various geographical regions and participates to the degradation of the hydrocarbons (Garcia-Junco et al., 2001; Norman et al., 2002; Kaszab et al., 2010). To our knowledge, no recent studies have been performed to better appreciate its relative abundance within the global soil bacterial communities, and to assess the influence of soil characteristics and anthropogenic constraints on its distribution. For instance the presence of *P. aeruginosa* in various water sources and in fecal material raises questions about the potential dispersion in soil through common agricultural practices i.e., irrigation and organic amendment, and then on the role of human activity for such dispersion. The presence of *P. aeruginosa* has been previously reported in both farmyard manure (Lavenir et al., 2007; Colinon et al., 2013) and composted industrial wastes (Kaszab et al., 2011). Human activities could also act as indirect selective pressures through the addition of chemicals i.e., pesticides, antibiotic, hydrocarbons known to be metabolized by *P. aeruginosa* and that could favor and enrich indigenous *P. aeruginosa* populations. This study is then dedicated to filling this knowledge gap.

Here we present a study investigating the prevalence of *P. aeruginosa* in agricultural soils as influenced by soil properties and the potential impact of agricultural practices on this prevalence. Culture-dependent and culture-independent (i.e., real-time quantitative PCR, qPCR) approaches were performed to quantify the occurrence of *P. aeruginosa.* More precisely we combined a previously developed SYBR Green qPCR assay targeting the *ecf*X gene (Colinon et al., 2013) and culturing of bacteria on a semi-selective media (nalidixic-supplemented cetrimide agar base) coupled to *ecf*X gene amplification to confirm isolate identity. *ecf*X encodes an ECF (extracytoplasmic function) sigma factor which is restricted to *P. aeruginosa* and was previously validated to improve both sensitivity and specificity of *P. aeruginosa* detection in environmental samples (Lavenir et al., 2007). Presented data are from a study conducted on a broad spatial scale using various soil types, under various land uses, and from various geographical origins, partly using the collection belonging to the French national soil survey (Arrouays et al., 2002). In addition we monitored the impact of various organic amendments, including cow and horse manure, sewage sludge, and composted or raw urban wastes, spread onto experimental fields in France and in Burkina Faso.

## Materials and methods

### Sources of French soil samples

**Figure 1:**
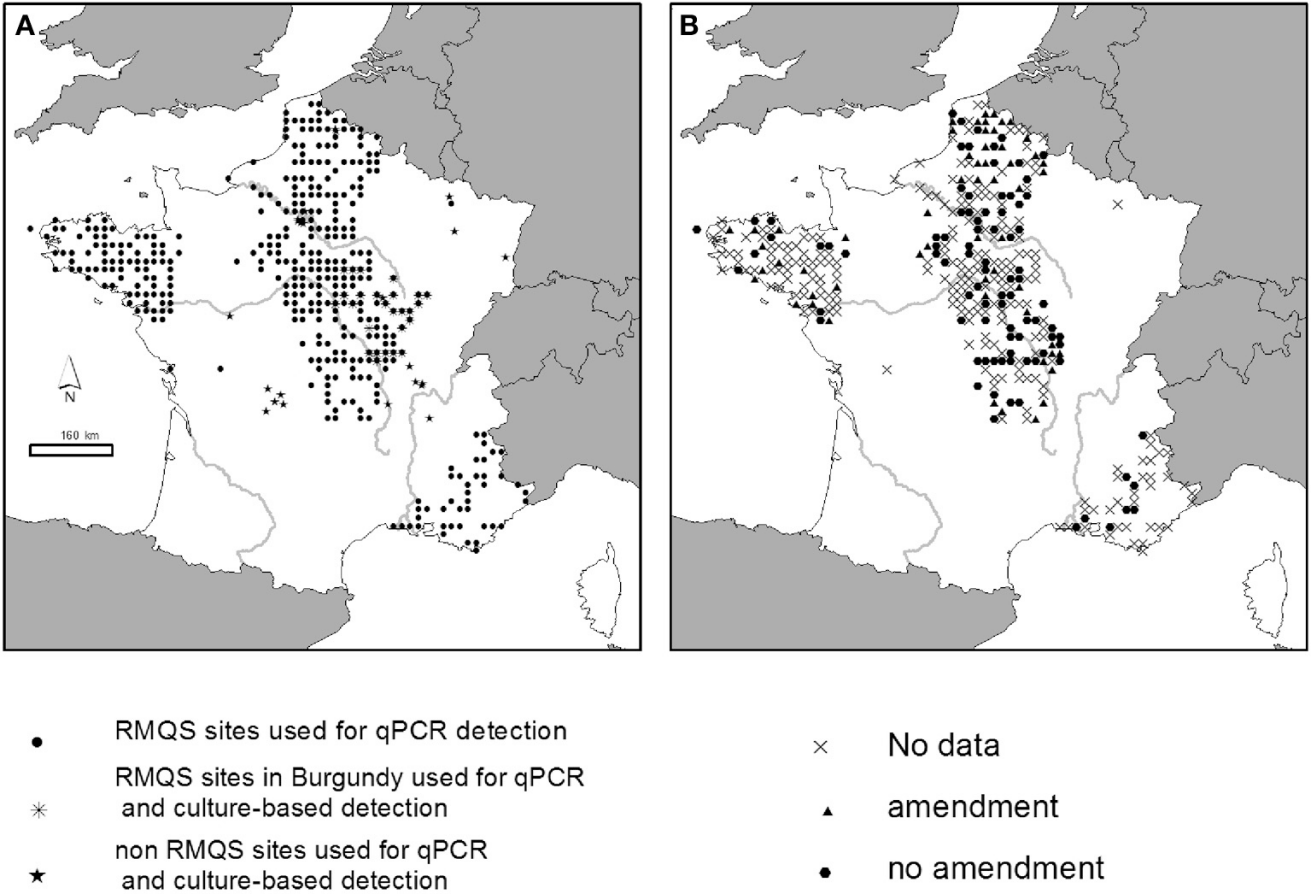
**Sites studied in France. (A)** Soils used for qPCR and culture-based detection of *P. aeruginosa*. **(B)** Soils that received or not organic amendment.

A set of 380 soil samples was selected within the French RMQS (“Réseau de Mesures de la Qualité des Sols” = French soil quality monitoring network) for the qPCR detection of *P. aeruginosa* (Figure 1A). The RMQS is a soil library which contains 2200 soils sampled with a 16 km × 16 km systematic grid over the whole French territory, and representative of the different soil types, land cover, land management type and climatic conditions occurring in France [for more details, see (Arrouays et al., 2002)]. The 380 RMQS samples were chosen from 5 different geographical regions (Brittany, South-East, North, Center, Bassin Parisien). These regions were chosen in order to get samples with various geographic, pedo-climatic, and land use characteristics, as well as exposure to various organic amendments. Sixty-three out of the 380 soils received repeated organic amendment over several years from various sources before sampling: bovine manure (*n* = 49), liquid swine manure (*n* = 10), poultry dropping (*n* = 1), or sewage sludge (*n* = 3) (Figure 1B). Ninety-seven out of 380 soils had no known history of amendment. This information was not available for the remaining soils (220 out of 380). Twenty-six locations from Burgundy within the Center region were selected among the 380 samples to search for the presence of *P. aeruginosa* using the culture-dependent approach. Additional soil samples were collected from various agricultural areas in France including Limousin, Burgundy, Rhône-Alpes, North, and Bassin Parisien regions (Figure 1A) and from 2 experimental sites from the INRA research center in Chinon (Center region) and Mâcon (Burgundy region). These agricultural soils were planted with maize, wheat or grapevine. During the year of collection, some soils received organic amendments mainly farmyard manure, and/or were exposed to animal grazing, others did not (Table 1). The samples, i.e., ten samplings per field composing one sample, were collected from the upper layer (0–5, 0–10 or 0–20 cm), sifted through 2 mm-mesh sieves and stored at room temperature for no longer than 1 week. They were collected during various campaigns between 2006 and 2011. Soil physico-chemical characteristics for some sites tested with qPCR- and culture-based approaches are presented in Table S1 to show the extent diversity of the soils studied.

**Table 1:** **Number of CFU (colony forming units) of *P. aeruginosa* in organic amendments and agricultural soils amended or not with organic wastes**.

**Region and city**	**Description at sampling time amendment or treatment during the year**	**Number of treated samples**	***P. aeruginosa* CFU × 10^3^ (g drywt sample)^−1^(±standard deviation)**
**ORGANIC AMENDMENTS**			
Alsace			
Colmar-INRA	Wet bovine manure	3	2.7 (±0.47)
	Wet compost of bovine manure	3	0
Bassin Parisien			
Versailles	Green waste and animal powder	2	0
	Poultry dropping	2	0
	Dried pig manure	2	0
	Compost of horse manure mixed with wood chip (farm 1)	2	0
	Compost of horse manure mixed with farm wheat straw (farm 2)	2	62 (±10)
	Compost of horse manure mixed with commercial wheat straw (farm 3)	2	164 (±18)
Feucherolles	1 month wet bovine manure (FYM) year 2006	3	15 (±2.2)^a^
	1 month wet bovine manure (FYM) year 2007	3	5.4 (±1.4)^a^
	Compost of municipal solid wastes (MSW)	2	0^−^
	Compost of fermentable fraction of municipal wastes and green wastes (BW)	2	0^−^
	Compost of sewage sludge, green wastes and wood chips (GWS)	2	0^−^
Rhone-Alpes			
Versailleux	Poultry dropping (farm 1)	3	0^−^
	6 months dry bovine manure (farm 1)	3	0
	1 month wet bovine manure (farm 1)	3	0
	1 month wet bovine manure (farm 2)	3	19 (±3.8)^a^
Joyeux	Months dry bovine manure	3	0
Saint Olive	1 month horse manure	3	5.5 (±1.1)^a^
**SOIL SAMPLES**			
Burgundy—RMQS library			
Balot	Barley, nd	2	0
Venarey les laumes	Grassland, animal grazing, no manure	2	0
Is sur Tille	Rapeseed, bovine manure	2	0^−^
Bourberain	Bourberain rapeseed, nd	2	0
Dompierre en Morvan	Grassland, no manure	2	0
Ruffey les Echirey	Rapeseed, vegetal compost, no manure	2	0
Marcilly Ogny	Triticale, bovine manure	2	0^−^
Commarin	Rapeseed, nd	2	0
Echevronne	Cereal, nd	2	0
Morey Saint Denis	Vineyard, nd	2	0
Saint Aubin	Vineyard, nd	2	0
Moulins-Engilbert	Grassland, animal grazing, bovine manure	2	0
Cudot	Rapeseed, nd	2	0^−^^a^
Joigny	Wheat, nd	2	0^−^^a^
Brienon sur Armancon	Maize, nd	2	0^−^^a^
Treigny	Grassland, animal grazing, no manure	2	0^−^^a^
Merry sur Yonne	Cereal, nd	2	0^−^^a^
Angely	Grassland, animal grazing	2	0^−^^a^
Saint Pere	Cereal, no manure	2	0^−^
Courcelles	Cereal, nd	2	0^−^
Gilly sur Loire	Grassland, animal grazing, nd	2	0^−^^a^
Rigny sur Arroux	Grassland, animal grazing, no manure	2	0^−^^a^
Palinges	Grassland, animal grazing, bovine manure	2	0^−^^a^
La Guiche	Grassland, animal grazing, bovine manure	2	0^−^^a^
Salornay sur Guye	Fallow, nd	2	0^−^^a^
L'Hopital le Mercier	Grassland, animal grazing, pig slurry	2	0^−^^a^
Bassin parisien			
Pierrelaye 1	Maize	10	0
Pierrelaye 2	Miscanthus, sewage sludge and waste water	10	0^−^
Pierrelaye 3	Maize, sewage sludge and waste water	21	0^−^
Chavenay	Wheat, composted horse manure from Versailles farms	3	0^−^
Fontenay le Fleury	Wheat, non-composted horse manure from Versailles farms	3	0^−^
Unknown site 1	Industrial site, hydrocarbon contamination	2	1.1 (±0.13)
Unknown site 2	Industrial site, hydrocarbon contamination	2	34 (±7.0)
Unknown site 3	Industrial site, hydrocarbon contamination	2	0^+^
Limousin			
St Iriex	Maize, farmyard manure	1	0
St Iriex	Grassland, animal grazing	1	0
Champnetery	Wheat, farmyard manure	1	0
Champnetery	Grassland, animal grazing	1	0
St Genest	Maize, farmyard manure	1	0
St Genest	Grassland, animal grazing	1	0
Sussac	Wheat, farmyard manure	1	0
Sussac	Grassland, animal grazing	1	0
Chaptelat	Forest	1	0^−^
Lorraine			
Neuves-Maisons^a^	Industrial site, hydrocarbon contamination	2	0.33 (±0.46)
Homecourt	Industrial site, hydrocarbon contamination	2	0^−^
North			
Courcelles-les-Lens 1	Miscanthus, heavy metal contamination	3	0^−^
Courcelles-les-Lens 2	Wheat, heavy metal contamination	3	0^−^
Dourges	Miscanthus	3	0^−^
Rhone-Alpes			
Versaileux	Maize, dry bovine manure from farm 2	3	0^−^
La Côte Saint-André	Maize	1	0^−^
Montrond	Grassland	1	0^−^
Burgundy—INRA site			
Mâcon	Vineyard	2	0^−^
Mâcon	Vineyard, mulching straw	2	0^−^
Mâcon	Vineyard, conifer compost	2	0^−^
Center—INRA site			
Chinon	Vineyard	2	0^−^
Chinon	Vineyard, mushroom manure	2	0^+^
Chinon	Vineyard, vine shoot	2	0^−^
Chinon	Vineyard, bovine manure	2	0^−^

− No P. aeruginosa detection after enrichment

+ P. aeruginosa detection after enrichment.

a Data previously reported in Colinon et al. (2013).

We also included soils from industrial sites contaminated with hydrocarbons in Bassin Parisien (soils kindly provided by Biogénie Europe SAS, Echarcon, France) and in the Lorraine region (soils kindly provided by Dr. C. Leyval, UMR CNRS 7137, Nancy, France).

### Sources of organic amendments

We included poultry dropping, 1 month or 6 months-old bovine and horse manures obtained from 5 farms in the Dombes area (Rhône-Alpes), 1 month-old bovine manure from Colmar (Alsace) as well as various organic amendments i.e., bovine manure, compost of horse manure, poultry dropping, dehydrated pig manure and various municipal composted wastes, that are used on the Feucherolles experimental site (Bassin Parisien; see below) (Table 2), or on various fields around Versailles (Bassin Parisien). Part of these amendments was provided by INRA of Grignon.

**Table 2:** **Culture-based detection of *P. aeruginosa* in soils exposed to various organic amendments**.

**Site, sampling year, amendment type**	**Number of treated samples**	**CAB CFU × 10^3^ (g drywt soil)^−1^ (±standard deviation)**	**CAB after enrichment *P. aeruginosa* detection (number of positive samples *per* treated sample)**
**FRANCE**			
Feucherolles—INRA site, 2006			
Before amendment			
Control	4	0	−
BW	4	0	−
FYM	4	0	−
GWS	4	0	−
MSW	4	0	−
1 month after amendment			
Control	4	0	−
BW	4	0	−
FYM	4	0	−
GWS	4	0	−
MSW	4	0	−
3 months after amendment			
Control	4	0	−
BW	4	0	−
FYM	4	0	−
GWS	4	0	−
MSW	4	0	−
**BURKINA FASO**			
Tabtenga			
2007, control	2	0	−
2007, UW	2	0	−
2008, control	3	0	−
2008, UW	3	0	−
2011, control	6	0	−
2011, UW	12	0.01^a^	+(4/12)
Toudoubweogo			
2007, control	2	0	−
2007, UW	2	0	−
2008, control	3	0	−
2008, UW	3	0	−
Zagtouli			
2008, control	3	0	−
2008, UW	3	0	−
Yagma			
2007, control	2	0	−
2007, UW	2	0	+(1/2)

BW: compost of fermentable fractions of municipal wastes and green wastes; FYM: bovine farmyard manure; GWS: compost of sewage sludge, green wastes and wood chips; MSW: compost of municipal solid wastes in France, and untreated urban waste (UW) amendment in Burkina Faso.

−Negative isolation after acetamide enrichment.

+Positive isolation after acetamide enrichment.

a Only 1 P. aeruginosa like-colony was detected on CAB plate spread with 1 ml of the undiluted soil suspension.

### Experimental sites with organic amendments in France and Burkina Faso

The site of Feucherolles was used for 8 years to assess the effects of urban composts on soil fertility and their environmental impact (Houot et al., 2002). The field experiment was cropped with a wheat-maize succession for the time the experiment lasted. Organic amendments included (1) a municipal solid waste compost (MSW) made from residual municipal wastes after selective collection of clean, dry packaging, (2) a biowaste compost (BW) made from selectively collected fermentable fractions of municipal wastes co-composted with green wastes, (3) a compost produced from the co-composting of sewage sludge, green wastes and wood chips (GWS), and (4) a farmyard manure (FYM) issued from cow breeding. These four organic treatments were compared to an organic input-free control. The experiment was a randomized complete-block designed with four replicates. Samples were collected from the upper layer (0–10 cm) in September (before amendments were added), October (1 month after amendments were added) and December (3 months after amendments were added) for two successive years (2006 and 2007). Each treatment was performed on four plot replicates and samples were made of a composite sampling (10 samplings per plot replicate). The culture approach was tested on all replicates, i.e., a total of 20 soil samples (4 replicates × 5 treatments) per sampling date.

Four sahelian sites (Tabtenga, Zagtouli, Yagma and Toubwéogo) located in the sub-urban area of Ouagadougou, Burkina Faso, were added to our study in order to include soil types from regions of different climate conditions from those observed in France. This also enabled us to assess the impact of the local farmers' practices, which differed from the French farmers' and involved the use of untreated domestic and urban wastes on agricultural fields. The sites in Burkina Faso were planted with sorghum and impacted by untreated wastes (UW) due to long-term practices (between 6 and 18 years) by local farmers. The amendments induced an increase in organic carbon content, and in pH values (Table S1). The samplings were performed during three campaigns conducted in March 2007, June 2008 and February 2011. At each site sampled in 2007 (Tabtenga, Toubwéogo, and Yagma) two transects per field were analyzed. In 2008, three transects per field were made at Tabtenga, Toubwéogo, and Zagtouli. At Tabtenga in 2011 one transect was made in the control plot and 6 samples were analyzed, while 3 transects were made in the amended field and 4 samples *per* transect were taken. At each site in 2007, 2008, and 2011 soils were sampled from the upper layer (0–5 cm) and the samples were made of a composite sampling (10 samplings over a 20 m transect). During the 2008 campaign additional soil samples from a bellowed horizon (5–20 cm) were also collected.

### Enumeration and identification of culturable *Pseudomonas aeruginosa*

*P. aeruginosa* enumeration was performed using Cetrimide Agar Base (CAB) medium (Oxoïd, Cambridge, UK) supplemented with nalidixic acid (15 mg l^−1^) and cycloheximide (200 mg l^−1^). Soil bacterial cells were extracted by blending 5 g of soil with 50 ml of a saline solution (NaCl 0.8%) for 90 s in a Warring Blender (Eberbach Corporation, Ann Arbor, MI, USA). The homogenized soil suspensions were serially diluted in sterile saline solution, and aliquots of the appropriate dilutions were spread onto agar plates. In all cases, three plates were inoculated per dilution and incubated at 28°C for up to 3 days. To ensure that 28°C would be the appropriate temperature to recover *P. aeruginosa* from environmental samples we performed preliminary tests and compared plating at 28 and 37°C for all soil samples from Burkina Faso and for some French soils including the industrial ones and some manure amendments. In parallel we also checked on more than 50 clinical and environmental strains from our own collection and from international collections whether strains grow differently at 28 and 37°C. For both tests results were similar whatever the temperature of incubation. We then decided to do further screening at 28°C. One hundred μl were usually spread *per* 90-mm diameter plate. To improve *P. aeruginosa* detection sensitivity, 1 ml of soil suspension were used for the soil samples from Burkina Faso as these soils contained high quantities of sand, low quantities of organic matter, and therefore, yielded clear soil suspensions. In addition, enrichment assays were performed by transferring 2 g of soil into 20 ml of a salt solution supplemented with acetamide as described previously (Green et al., 1974). Inoculated enrichment broths were incubated for 3 days at 28°C with shaking at 180 rpm. Serial dilutions were performed and plated on CAB agar medium.

All the greenish and clearly yellowish colonies were collected from CAB plates and confirmed as *P. aeruginosa* by PCR screening with the *ecf*X gene encoding for an ECF (extracytoplasmic function) sigma factor, as previously described (Colinon et al., 2013) and by oxidase assay. All *ecf*X- and oxidase-positive isolates were submitted to partial 16S rDNA sequencing to confirm their identification. The detection limit using cultivation-based measurements varied from 10 to 100 CFU (g drywt soil)^−1^ (Colinon et al., 2013).

As our study evaluated the impact of amendment but that samplings were mostly performed long after amendment i.e., at least a month, we set up a laboratory experiment to evaluate how long cells could survive and be detected in soils using the culture approach. An agricultural soil from a field planted with maize (La Côte Saint André, Rhône-Alpes region; Table 1) was chosen to conduct that experiment (Table 1). A clinical strain PAO1 and an environmental strain ATCC 31479 were added at a concentration of 1.2 × 10^6^ and 1.8 × 10^6^ CFU (g drywt)^−1^ of soil, respectively. Microcosms of 10 g of sieved (2 mm) soil were inoculated with cell-water suspensions. Microcosms were incubated at 20°C for 38 days. They were set up in triplicates and were sacrified at each time point (day 3, 6, 16, 24, 38). The same experiment was conducted on sterilized soil from La Côte Saint André (gamma irradiation sterilization; Ionisos, Dagneux, France). Microcosms were inoculated with PAO1 and ATCC 31479 at levels of 1.09 x 10^8^ and 1.42 x 10^8^ CFU (g drywt)^−1^ of soil, respectively. Microcosms were set up in triplicates and were analyzed at each time point (2, 6, 17, 44, and 58).

### DNA extraction and purification

The isolated strain genomic DNA was extracted by gentle alkaline lysis (using sarkosyl and NaCl) and purified according to Johnson (1994), and DNA concentration was estimated using a Nanodrop^®^ ND-1000 spectrophotometer (Labtech International, Paris, France) at a 260 nm wavelength. DNA from soils of the RMQS grid were extracted by the GenoSol platform at INRA Dijon according to a single procedure optimized by Ranjard et al. (2003). DNA from soils sampled in Pierrelaye, Feucherolles and Burkina Faso and amendment samples (0.5 g) were extracted in triplicates with the FastDNA^®^ SPIN Kit for Soil (MP Biomedicals, Solon, OH, USA) and purified on S-400-HR mini-columns (Pharmacia, St Quentin Yvelines, France) following the manufacturer's instructions. The DNA extracts were resolved by electrophoresis in 0.8% agarose gels, stained with ethidium bromide and photographed using a Gel Doc 1000 camera (Bio-Rad, Ivry sur Seine, France). DNA concentrations in the crude and purified soil extracts were determined by electrophoretic comparison with a standard curve as previously described (Ranjard et al., 2003).

### *Pseudomonas aeruginosa*-specific qPCR for soil samples

*Pseudomonas aeruginosa*-specific qPCR was set up using the ECF5 (5′-AAGCGTTCGTCCTGCACAA-3′) and ECF2 (5′-TCATCCTTCGCCTCCCTG-3′) primers. These primers amplify a 146-bp long fragment of the *ecf*X gene (Colinon et al., 2013). The Eva Green SMX 1000R (Bio-Rad) was used, according to the manufacturer's instructions. The reactions were carried out in a 20-μl reaction mixture containing 500 nmol l^−1^ of each primer. Non-template controls, including the reaction mixture with sterile water instead of DNA template, were added to each run. Quantitative PCR was performed in a LightCycler 480 system (Roche Diagnostics, Meylan, France). The following PCR protocol was applied: initial denaturation at 95°C for 5 min, followed by 45 cycles with denaturation at 98°C for 10 s, annealing and elongation at 63°C for 20 s. Subsequently a melting curve was recorded by increasing the temperature from 65 to 98°C (+1°C every 10 s). For reproducibility, all qPCRs were triplicated on separate plates. *P. aeruginosa* strain UCBPP-PA14 genomic DNA was used to generate a standard curve. Quantification was performed by comparison with a 2- to 5-fold diluted standard (1 fg to 100 pg, 5 μl *per* reaction). Data analysis was performed using the LightCycler^®^ 480 software (Roche Diagnostics). PCR product specificity was checked by melting curve analysis and agarose gel electrophoresis. Based on the results of previous optimization tests (Colinon et al., 2013), 5 ng of soil DNA extracts (5 μl each *per* reaction) were screened in triplicates using 25 ng μl^−1^ of T4 Gene 32 protein. qPCR results were interpreted as undoubtedly positive only when the fluorescence signal obtained corresponded to at least 50 fg of *P. aeruginosa* cells (7 genome equivalent) at the appropriate corresponding Tm.

Sensitivity of the qPCR approach was determined to be around 5 × 10^4^ cells (g drywt soil)^−1^ (Colinon et al., 2013).

### Control tests for DNA quality and *P. aeruginosa* DNA persistance

The quality of genomic DNA is critical for success of the qPCR. Before being screened for the presence of *P. aeruginosa* we then checked for the presence of inhibitors in DNA extracts and their impact on our ability to amplify added DNA target or intrinsic DNA target. Two tests were then performed. In the first one we added 10^6^ copies of the circularized pGEM-T Easy plasmid DNA (2.5 μl *per* 20 μl of PCR reaction) to 5 ng of each DNA. The plasmid DNA was quantified afterwards by comparison with a 10-fold diluted standard of pGEM-T Easy plasmid DNA (10^1^ to 10^7^ copies, 2.5 μl each *per* reaction). qPCR reactions and cycling conditions were as stated above, except for the primers we used which were SP6 and T7 universal primers, the annealing temperature which was set at 55°C, and the PCR kit which was the LightCycler§ 480 SYBR Green I Master kit (Roche Diagnostics). A 10-fold reduction of the copy number amplified by qPCR with respect to the copy number introduced in the reaction mixture was considered as 10% amplification efficiency, or, conversely, as 90% inhibition.

In the second test we performed a 16S rRNA primer-based assay as described in previous studies (Lopez-Gutierrez et al., 2004). Samples were screened once using 2 ng of template DNA. The assay was only conducted on RMQS DNA extracts.

## Results

### Detection of *P. aeruginosa* in organic amendments

The culture dependent approach was performed on various organic materials including poultry dropping, animal manures of various ages and humidity content, composted or not, and various composts of municipal wastes (Table 1). No *P. aeruginosa* were isolated from poultry dropping, dehydrated pig manure and the mix of green waste and animal powder samples. Analysis of manures collected from farms in Bassin Parisien, Rhône-Alpes and Alsace showed the presence of *P. aeruginosa* at levels of 5.5 (±1.1) × 10^3^ to 164 (±18) × 10^3^ CFU (g drywt)^−1^ for horse manure and at level of 2.7 (±0.47) × 10^3^ to 19 (±3.8) × 10^3^ CFU (g drywt)^−1^ for bovine manure. It has to be noted that *P. aeruginosa* was not isolated from 6 months-old bovine manure and was still detected after a compost treatment of horse manure. qPCR enabled quantification of *P. aeruginosa* at level of 23 (±3.5) × 10^3^ and 76 (±15) × 10^3^ cells (g drywt)^−1^ in the manure samples used at the Feucherolles site in 2006 and 2007, respectively, as reported in our previous study (Colinon et al., 2013). Here we performed qPCR on DNA extracts from manure samples collected in Rhône-Alpes and Alsace. In these samples *P. aeruginosa* was detected at a level of 28 (±7.5) × 10^3^ and 43 (±9.7) × 10^3^ cells (g drywt)^−1^ in the horse manure samples from St-Olive and the bovine manure from Versailleux, respectively. Positive results were confirmed by sequencing amplified products (data not shown). qPCR did not allow detection of *P. aeruginosa* in the other samples.

Analysis of the 3 composts derived from municipal wastes or sewage sludge used on the plots of the experimental site of Feucherolles did not indicate the presence of *P. aeruginosa*. Neither the culture approach nor the qPCR enabled detection of *P. aeruginosa* in these samples.

### Screening of soil DNA for the presence of *P. aeruginosa* using qPCR

Five percent of the 380 RMQS soil DNAs showed inhibition on qPCR above 10%. None of the DNA samples from Feucherolles, Pierrelaye and Burkina Faso soil samples showed significant inhibition (<1% inhibition). The ability to amplify indigenous DNA target was also tested using universal primers to detect 16rDNA sequences among the RMQS DNA extracts. The 16S rRNA gene copy numbers of bacteria ranged from 6.31 × 10^5^ to 1.02 × 10^9^ copies (g drywt)^−1^ in 33 out of 380 DNA samples and from 1.02 × 10^9^ to 1.06 × 10^11^ copies (g drywt)^−1^ of soil in the remaining DNAs.

All DNA samples were analyzed whether or not inhibition and differences in 16S rDNA copies were observed.

The survey of *P. aeruginosa* distribution based on the qPCR detection showed that none of the 380 DNA samples of the RMQS soil set emitted a noteworthy positive fluorescence signal. Similarly none of the samples from the Feucherolles, the Pierrelaye and the Burkina Faso sites gave a positive signal. We previously estimated the detection limit to be 5 × 10^4^ cells (g drywt)^−1^ of soil (Colinon et al., 2013). Then the absence of amplification from soil DNA extracts indicated that either *P. aeruginosa* is not present within these indigenous soil bacterial communities or is present at a low level, below the detection threshold of this assay.

### Detection of *P. aeruginosa* in soil based on culture dependent approach

To confirm or not the absence of *P. aeruginosa* in the RMQS soil samples we screened a subset of the RMQS library including 26 soils from the Burgundy region, using the culture-dependent approach (Table 1). To avoid changes in bacterial community composition or in physiological cell properties, all these samples were processed directly after being sampled and had not been dried and stored as were the samples from the RMQS library used for DNA extraction. The results showed an absence of *P. aeruginosa* even after an enrichment step. Similarly, detection with the culture dependent method was mostly unsuccessful with the agricultural soils sampled in other French regions including Bassin Parisien, Limousin, Lorraine, North, and Rhône-Alpes (Table 1). As observed in the control soil, direct isolation of *P. aeruginosa* from the organic-amended soils of the 2 INRA sites at Mâcon and Chinon was unsuccessful whatever the amendments. However the enrichment procedure enabled us to isolate *P. aeruginosa* from the vineyard soil amended with mushroom manure in Chinon (Table 1).

Despite the presence of *P. aeruginosa* in the studied horse manure-containing amendments (composts of horse manure from farms in Versailles) no *P. aeruginosa* was recovered from the soils that had previously received amendments originating from these farms [i.e., soils from Fontenay le Fleury and Chavenay (Bassin Parisien)] (Table 1). Detection was also unsuccessful with samples from soils that received either bovine manure or pig slurry, or that were exposed to animal grazing in Limousin and Burgundy.

It has to be noticed that negative results were also obtained with soil samples from agricultural fields either planted with maize and miscanthus which received sewage sludge and were irrigated for more than 100 years with untreated and further treated urban wastewater (soils from Pierrelaye, Bassin Parisien). We expected these soils to contain *P. aeruginosa* as that species is frequently detected in wastewaters (Lavenir et al., 2007; Lee et al., 2008). As a comparison and because of the literature reports about the presence of *P. aeruginosa* in environments contaminated with hydrocarbons, we included some soil samples collected from hydrocarbon-polluted industrial sites. Two out of the 3 soils from Bassin Parisien and one located in Lorraine (soil from Neuves-Maisons) led to the direct isolation of *P. aeruginosa* on selective medium. Statistically different counts were observed as the numbers of CFU were 1.1 (±0.13) × 10^3^ (*p* value = 0.0013) 34 (±7.0) × 10^3^ (*p* value = 0.00022), and 0.33 (±0.46) × 10^3^ (*p* value = 0.0015) (g drywt)^−1^ in the three soils, respectively. The enrichment step with acetamide enabled us to isolate *P. aeruginosa* in the third sample from Bassin Parisien but not in the sample from the Homécourt soil in Lorraine.

### Temporal impact of organic amendments on *P. aeruginosa* dissemination at experimental sites

The experiment conducted to evaluate whether *P. aeruginosa* can still be detected by plate-counting after addition in soil showed that both strains were still detectable up to day 58 (Table 2) in sterilized microcosms. Their level was still high reaching 2.9 (±2.3) × 10^7^ and 1.3 (±0.67) × 10^7^ for PA01 and ATCC 31479, respectively. On the opposite the level of both strains declined in the non-sterile microcosm. PAO1 and ATCC 31479 were not detectable after 24 and 38 days, respectively (supplementary data; Table S2).

At the experimental site of Feucherolles, samples collected before amendment, 1 month and 3 months after amendment, did not led to the detection of *P. aeruginosa* whatever the amendment used and despite the presence of *P. aeruginosa* in the farmyard manure (Table 2) during year 2006. Detection was unsuccessful with or without adding the enrichment step in the isolation procedure. As the experiment was conducted again in 2007, soils were analyzed following the same sampling time scale. Similarly no *P. aeruginosa* was isolated.

The presence of *P. aeruginosa* was also investigated in soils amended with untreated urban wastes from 4 agricultural sites in the vicinity of Ouagadougou, Burkina Faso (Table 2). No *P. aeruginosa* was detected in the unamended soils whatever the site and the sampling year. None of the samples from the upper soil layer, except 1 from the site of Tabtenga from the 2011 sampling campaign, led to a direct isolation of *P. aeruginosa*. The positive sample enabled us to obtain only 1 colony. This sample was analyzed 3 times and either no or 1 colony was obtained. Then the estimation of *P. aeruginosa* abundance was below 10 CFU g^−1^ dry soil. Our enrichment assays led to the detection of *P. aeruginosa* in 1 soil sample from the Yagma site, and in 4 out of 12 samples collected in the Tabtenga amended field during the 2011 campaign. None of the samples from the 5–20 cm layer led to the detection of *P. aeruginosa*.

A genetic typing of some isolates obtained from the direct isolation and the enrichment procedure was performed using Multiple Locus Variable Number of Tandem Repeat (VNTR) Analysis (MLVA). Data showed that those isolates had different MLVA types and differed from other environmental and clinical isolates (Youenou et al., 2014) suggesting that the detection of *P. aeruginosa* in the soil samples from Burkina Faso was not due to a laboratory contamination.

## Discussion

In this study we conducted a survey of the prevalence of *P. aeruginosa* in French agricultural soils using both a culture-based approach and a culture-independent one to evaluate the capacity of soil environment to constitute a reservoir or a transient recipient of *P. aeruginosa*. This study intended to gain new insights on the distribution of *P. aeruginosa* in non-aquatic environments. Ringen and Drake (1952) addressed this point and observed that *P. aeruginosa* occurred in only 3 out of 100 soil samples. Recently through a synthesis of available literature Selezska et al. (2012) concluded that “*P. aeruginosa* is obviously an aquatic rather than soil organism” and as stated by Ringen and Drake (1952) the “isolations of *P. aeruginosa* from […] soil seem to be one of chance.” Despite of their different abiotic and biotic properties, land use and geographical origin, none among the wide range of the agricultural soils we selected in the RMQS soil library enabled the detection of *P. aeruginosa* based on the qPCR approach suggesting that none of these environmental conditions sustain this species growth. However *P. aeruginosa* could be present at a low abundance below the detection limit of our qPCR approach. Based on the culture dependent approach none of the RMQS sites and other French agricultural fields led to the isolation of *P. aeruginosa* with or without a previous enrichment procedure. These observations are consistent with the conclusions of Selezska et al. (2012), but contrast with previous reports from Green et al. (1974) who recovered it from 24% of the Californian soils they tested. However it has to be noted that they used an enrichment step with acetamide and could not isolate *P. aeruginosa* without it. A recent study on Hungarian compost-amended soils also mentioned the presence of *P. aeruginosa* but at a low level with counts between 10^0^ and 10^2^ MPN (Most Probable Number) per g of soil (Kaszab et al., 2011). In our work, we failed to recover isolates from French agricultural samples, suggesting that if soil is a natural habitat for *P. aeruginosa* then it is a minor population or that it colonizes specific soil niches (rhizosphere, macrofauna) not investigated in the present study.

The widespread presence of *P. aeruginosa* in various water sources (Khan et al., 2007; Lavenir et al., 2007; Lee et al., 2008), its carriage by human and animals (Lavenir et al., 2008; Szmolka et al., 2012) and its presence in compost manure (Edrington et al., 2009) raised the question of the role of anthropogenic activities on *P. aeruginosa* dissemination. We looked for the presence of *P. aeruginosa* in various organic amendments derived from animal farms as spreading bovine manure, pig slurry or poultry dropping are common French farmer practices to enrich soil in organic matter. We could not isolate *P. aeruginosa* directly from fresh horse and bovine feces on 6 tested samples from Versailleux and St Olive (data not shown) but confirmed its presence in horse and bovine manures collected from geographically distant farms in France (Bassin Parisien and Rhône-Alpes). Similarly Kaszab et al. (2011), previously reported the lack of detection of *P. aeruginosa* in various raw wastes except wheat straw whereas they were able to detect it after composting treatments. Interestingly we could not isolate this species from 6 months-old manure. As these samples were relatively dry (5% water content) compared to the 1 month-old manure (at least 50% water content) this observation suggest that dehydration could eliminate or lower *P. aeruginosa* abundance. This observation is in agreement with the hypothesis that *P. aeruginosa* preferentially inhabit wet environments. Our data also showed that compost of horse manure still contained *P. aeruginosa* cells whereas composting is a treatment used to lower the amount of pathogens in organic wastes. This observation suggests that the heat treatment used in our composting process (50°C for 4 weeks) does not eliminate *P. aeruginosa* and that *P. aeruginosa* tolerates changes in temperature. Increase in temperature might enable it to replicate during composting and might not affect its culturability, as previously reported (Kaszab et al., 2011).

Based on the observed abundance in the various manure samples tested in this study (i.e., about 10^4^ CFU g^−1^ dry soil) and on the mean amount of added manure to soil about 1 to 20 ton (ha year)^−1^ as indicated by farmers in the RMQS inquiry) we estimated the amount of *P. aeruginosa* introduced into soil to be about 1 × 10^10^ to 20 × 10^10^ cells (ha year)^−1^. These amounts are added in the first 20–30 cm once a year or every 2 years as usually done by French farmers. The RMQS library gave us the opportunity to screen 63 soils that were known to receive various amount and sources of organic amendments. None of the 49 and 10 soils that received bovine manure or pig slurry, respectively, enabled detection of *P. aeruginosa*. Similarly, most of the non-RMQS soil samples that received manure or that were used for animal grazing led to the detection of *P. aeruginosa*. The only samples in which *P. aeruginosa* was detected were collected from a mushroom manure-amended vineyard site (Chinon) in France and from urban waste-amended fields (Yagma and Tabtenga sites) in Burkina Faso. However the abundance of *P. aeruginosa* was very low: few isolates were successfully obtained from the Chinon and the Burkina Faso sites. Furthermore these isolates were mainly obtained after an enrichment procedure. The soil in Chinon presented the distinctive feature of being amended with mushroom manure. Unfortunately, at the time of sampling we could not obtain mushroom manure samples to check whether manure was the source of *P. aeruginosa* in the soil or whether *P. aeruginosa* was an autochtonous member of the soil microflora. *P. aeruginosa* was not isolated from the control soil. We then hypothesized that *P. aeruginosa* was present in the amendment because mushroom manure contains horse manure and *P. aeruginosa* was shown from this study and from a previous report (Lavenir et al., 2007) to be present in horse manure. The capacity of *P. aeruginosa* to persist in manure and then in amended soils has not been reported yet, but such persistence had already been demonstrated for other pathogenic bacteria (Pell, 1997; Gerba and Smith, 2005). In our study, the experimental site of Feucherolles gave us the opportunity to investigate the survival of *P. aeruginosa* in soils after various organic amendments were applied, including farmyard manure and compost of municipal wastes. Despite the presence of *P. aeruginosa* in the manure added in 2006 and 2007, we failed to recover it in the soil samples from the manure-amended plots 1 and 3 months after amendments were spread. These results were obtained for 2 years, consecutively. Our observations then suggested that *P. aeruginosa* was not indigenous to the bacterial community of that soil, (no *P. aeruginosa* was detected in the control soil), and that it may not survive easily in soils [no *P. aeruginosa* was detected in the manure-amended field, probably due to antagonistic interactions with the indigenous microflora (predation, substrate competition) or unfavorable physico-chemical soil properties]. Our very preliminary data on *P. aeruginosa* survival in soil suggested that exogenous cells added to soil can adapt to soil abiotic conditions but its persistence is affected by indigenous microflora. Identification of the soil parameters influencing *P. aeruginosa* survival should be investigated further also taking into account strain origin (human or animal) and intrinsic characteristics (i.e., antibiotic resistance properties) as well as the nature of *P. aeruginosa* sources (waste water, organic amendment. However we cannot rule out that *P. aeruginosa* survived whatever the conditions but became non-culturable, as in aquatic environments (Khan et al., 2010).

The presence of *P. aeruginosa* was also investigated in soils amended with untreated urban wastes in Burkina Faso. Despite long-term amendment practices (from 6 to 20 years), *P. aeruginosa* was only found in 2007 in an amended field in Yagma and in 2011 in an amended field in Tabtenga. It should be noted that few samples were positive *per* site and the level of *P. aeruginosa* was found lower than 10 cells *per* g of soil. We expected to detect it given that untreated wastes can contain potential pathogenic bacteria such as *Salmonella* or *E. coli* in a higher density than treated amendments, and fecal coliforms and streptococci, which indicate fecal contamination, frequently reach a density of 10^7^–10^8^ CFU g^−1^ dry soil (around 10^3^ in treated composts) (Deportes et al., 1998; Hassen et al., 2001). Moreover, *P. aeruginosa* has already been found in a Nigerian soil amended with urban waste (Achudume and Olawale, 2009). The fact that *P. aeruginosa* was not detected in the unamended fields whatever the site suggests that the amendment was at the origin of the presence of *P. aeruginosa*. Amendments can be a direct source of *P. aeruginosa* when wastes contain pathogens and do not undergo any treatment to eliminate them. They can also act indirectly: we observed that the use of organic amendments in fields increased total biomass, as well as the total number of heterotrophic bacteria (data not shown). Tracking *P. aeruginosa* into these soils is made difficult due to the complex quality and the combined origins of these untreated wastes that are made of domestic, animal and hospital wastes. However, due to the potential pathogenicity of *P. aeruginosa* and its innate resistance to antimicrobial agents, these wastes would need to be treated before being applied to soils in order to avoid human infection. The treatment has to be suitable for this pathogen as our study and previous reports (Kaszab et al., 2011) showed that *P. aeruginosa* is not eliminated and could be enriched during composting.

An intriguing observation is the frequent isolation of *P. aeruginosa* from hydrocarbon contaminated sites both aquatic (Bartha, 1977; Bhattacharya et al., 2000) and terrestrial (Norman et al., 2002; Kaszab et al., 2010) hydrocarbon-impacted environments. Despite the low number of samples tested our detection was very successful since *P. aeruginosa* was detected in 4 out of the 5 samples. This observation raises the question of its origin in these soils: indigenous soil population or exogenous population added with the hydrocarbons. Similarly we can wonder the role of *P. aeruginosa* intrinsic properties in its ability to opportunistically take advantages of contamination. In such environments *P. aeruginosa* can degrade hydrocarbons and/or act as a helper population that facilitates accessibility to hydrocarbons due to the synthesis of biosurfactants (i.e., rhamnolipids) (Garcia-Junco et al., 2001). Its ability to synthesize pyocyanin an inhibitor of other bacterial species, may also increase its competitiveness and survival in oil-contaminated sites (Norman et al., 2002).

We evidenced that *P. aeruginosa* is very rarely present in soils from agricultural lands and that recovery is more likely from environments exposed to intense anthropogenic activities, i.e., soils amended with untreated wastes in the tropical context or exposed to hydrocarbon contamination. We then concluded that soils are not reservoir for this species and that the encountered biotic and abiotic conditions may not favor its growth and spread. In some circumstances, i.e., the use of organic amendments, it was isolated from a few soil samples but always at a very low level suggesting that the risk for community-acquired infections among humans might be low. However the duration of its persistence and the environmental conditions and/or the alternative soil niches (plant roots, soil macrofauna) that could favor its persistence need further investigations.

### Conflict of interest statement

The authors declare that the research was conducted in the absence of any commercial or financial relationships that could be construed as a potential conflict of interest.
