# Supplementary material for: Low occurrence of Pseudomonas aeruginosa in agricultural soils with and without organic amendment
Source: Front Cell Infect Microbiol. 2014 Apr 29;4:53. doi: 10.3389/fcimb.2014.00053 (PMC4010769; doi:10.3389/fcimb.2014.00053)
Supplement: Supplementary file 1 [file DataSheet1.PDF]

Table S1: Soil physico-chemical characteristics of a subset of sites

Sites	Depth (cm)	Clay (%)	Silt (%)	Sand (%)	Corg g.kg-1	Norg g.kg-1	pH(H_2O)	C.E.C.[a] mEq.100g-1
Burkina faso								
Tabtenga Control	0-5	78	185	737	2.997	0.263	5.8	2.21
Tabtenga UW	0-5	59	172	769	12.47	1.29	7.3	4.55
Toubwéogo Control	0-5	73	314	613	11.57	0.512	7.1	2.93
Toubwéogo UW	0-5	114	263	623	29.33	2.13	8.1	8.01
Zagtouli Control	0-5	72	218	710	11.27	0.529	6.5	3.37
Zagtouli UW	0-5	107	214	679	72.03	2.67	8.1	11.17
Yagma Control	0-5	88	184	728	5.4	0.367	6.2	3.32
Yagma UW	0-5	103	191	706	28.1	1.53	8.2	7.6
Ile de France								
Feucherolles	0-20	150	783	67	11	1	6.9	9.4
Pierrelaye 1	0-10	70	89	841	9.9	0.821	7.5	4.69
Pierrelaye 2	0-10	71	101	830	15.45	1.02	6.7	5.01
Pierrelaye 3	0-10	80	120	800	20.98	1.51	6.7	6.31
Burgundy								
Balot	0-20	289	689	22	12.6	1.29	5.8	9.84
Venarey les Laumes	0-20	437	523	40	22.5	2.33	6.8	21.7
Is surTille	0-20	444	478	78	25	2.65	8.1	31.6
Bourberain	0-20	390	583	27	18.8	1.83	7	21
Dompierre en Morvan	0-20	180	457	363	23.7	2.27	5.6	6.53
Ruffey les Echirey	0-20	468	388	144	24.7	2.25	8.1	29.3
Marcilly Ogny	0-20	360	483	157	33	3.21	7.9	32
Commarin	0-20	421	499	80	22.1	2.4	7.1	19.5
Echevronne	0-20	448	414	138	34.5	3.37	7.9	32.2
Morey Saint Denis	0-20	334	427	239	11.6	1	8.3	20
Saint Aubin	0-20	345	424	231	12.2	1.12	8.2	17.8
Moulins Engilbert	0-20	552	384	64	29.2	2.93	6.9	24.3
Cudot	0-20	215	522	263	9.84	1.03	8.2	13.2
Joigny	0-20	321	303	376	19.2	1.77	8	16.6
Brienon sur Armancon	0-20	583	343	74	26.4	2.81	8	37.8
Treigny	0-20	229	401	370	25	2.43	5.9	11.6
Merry sur Yonne	0-20	438	454	108	27.5	2.78	8.1	29.5
Angely	0-20	506	470	24	38.1	3.66	5.9	23.6
Saint Père	0-20	345	412	243	15.09	1.56	5.9	13.79
Courcelles	0-20	333	409	258	13.55	1.27	5.8	7.75
Gily sur Loire	0-20	78	104	818	9.83	0.997	5.9	3.37
Rigny sur Arroux	0-20	158	685	157	14.00	1.23	5.4	3.67
Palinges	0-20	246	542	212	25.60	2.51	4.9	5.26
La Guiche	0-20	479	357	164	36.62	3.53	6.2	21.12
Salornay sur Guye	0-20	514	364	122	25.59	2.53	7.8	27.44
L'Hopital le Mercier	0-20	110	214	676	14.77	1.55	6.0	7.81

[a]C.E.C. Cation Exchange Capacity

Sites	Cd	Cu	Pb	Zn	Cr	Ni
	mg.kg-1	mg.kg-1	mg.kg-1	mg.kg-1	mg.kg-1	mg.kg-1
Burkina faso						
Tabtenga Control	0.38	5.65	6.33	17.3	39.1	7.41
Tabtenga UW	0.559	13.9	11	87.5	40.8	7.97
Toubwéogo Control	0.523	10.9	14.5	40.3	44	8.94
Toubwéogo UW	0.833	38.6	117	242	63.5	13.9
Zagtouli Control	0.428	10.1	10.3	18.9	56.7	13.1
Zagtouli UW	1.17	59.5	363	770	80.3	20.1
Yagma Control	0.536	31.9	11.7	88.4	49.5	10.5
Yagma UW	0.576	37.8	23.6	152	59	13.4
Ile de France						
Feucherolles	0.23	11.6	25.3	49.2	45.3	15.6
Pierrelaye 1	0.42	10.1	20.9	42.4	39.5	9.28
Pierrelaye 2	1.06	138	223	401	72	16
Pierrelaye 3	6.14	247	428	826	134	24
Burgundy						
Balot	0.352	13.1	38.7	86	70	32.7
Venarey les Laumes	0.284	23.8	29.7	141	90	48.8
Is surTille	0.722	22.1	31.4	121	94.5	41.7
Bourberain	0.539	17.3	30	85.3	87.6	36.9
Dompierre en Morvan	0.369	10.8	126	181	39	16.4
Ruffey les Echirey	0.503	30.3	48.5	112	75	36
Marcilly Ogny	0.974	16.5	38.7	146	122	57.9
Commarin	0.256	23.5	33.1	168	111	56.4
Echevronne	0.555	12.6	35.2	73	80.6	31.5
Morey Saint Denis	0.411	217	21.3	88.3	67.6	27.8
Saint Aubin	0.469	161	21.3	86.7	55.8	24.9
Moulins Engilbert	0.265	15.7	37.1	75.4	91	43.3
Cudot	0.249	8.06	22.8	41.5	40.2	14.3
Joigny	0.344	22.1	43.3	91.1	57	23.6
Brienon sur Armancon	0.311	24.2	38.6	151	96.5	47.9
Treigny	0.164	10.1	21.9	52.5	43	15.4
Merry sur Yonne	1.11	13.4	34.7	110	87.6	39
Angely	0.145	19.1	27.4	125	118	48.8
Saint Père	0.28	12.9	40.3	64.3	59.6	27.5
Courcelles	1.06	15.2	50.2	105	91.8	47.6
Gily sur Loire	0.09	6.5	37.6	34.8	18.1	8.37
Rigny sur Arroux	0.07	5.91	33.9	34.5	40.8	9.86
Palinges	0.25	9.91	52.1	83.9	41.1	16
La Guiche	5.53	19.7	197	1231	50.4	30.6
Salornay sur Guye	0.5	20.1	39.3	112	80.4	48.7
L'Hopital le Mercier	0.13	8.5	66.6	48.3	26.9	8.72
