# Supplementary material for: Low occurrence of Pseudomonas aeruginosa in agricultural soils with and without organic amendment
Source: Front Cell Infect Microbiol. 2014 Apr 29;4:53. doi: 10.3389/fcimb.2014.00053 (PMC4010769; doi:10.3389/fcimb.2014.00053)
Supplement: Supplementary file 2 [file DataSheet2.PDF]

Table S2 : Culture-based detection of *Pseudomonas aeruginosa* strains after inoculation in microcosms of sterilized or non sterilized soil from La Côte Saint André (Rhône-Alpes, France). Data are the mean of counts from triplicate microcosms.

	sterilized soil CFU x 10^7 (g drywt soil)$^{-1}$ (± standard deviation)			non sterilized soil CFU x 10^6 (g drywt soil)$^{-1}$ (± standard deviation)	
Days	PAO1	ATCC31479	Days	PAO1	ATCC31479
2	3.9 (± 1.2)	6.9 (± 2.6)	1	4.4 (± 1.3)	2.5 (± 2.80)
6	3.4 (± 1.9)	6.1 (± 1.9)	3	0.85 (± 0.38)	0.45 (± 0.79)
17	2.8 (± 1.2)	3.6 (± 1.6)	6	0.12 (± 0.79)	0.39 (± 8.33)
44	3.6 (± 3.4)	1.7 (± 1.1)	16	0.035 (± 0.022)	0.67 (± 0.14)
58	2.0 (± 2.3)	1.3 (± 0.67)	24	0	0.027 (0.089)
			38	0	0
